# Porocarcinoma From Preexisting Hidroacanthoma Simplex: Dermoscopic Findings

**DOI:** 10.5826/dpc.1002a36

**Published:** 2020-04-20

**Authors:** Vincenzo Maione, Martina Perantoni, Enzo Errichetti, Tiziana Borra, Piergiacomo Calzavara Pinton

**Affiliations:** 1 Department of Dermatology, ASST Spedali Civili di Brescia, Brescia, Italy; 2 Institute of Dermatology, University of Udine, Department of Experimental & Clinical Medicine, Udine, Italy; 3 Department of Pathology, ASST Spedali Civili di Brescia, Brescia, Italy; 4 University of Brescia, Faculty of Medicine and Surgery, Brescia, Italy

**Keywords:** porocarcinoma, hidroacanthoma simplex, dermoscopy

## Introduction

Porocarcinoma (PC) is a malignant adnexal tumor that may develop “de novo” or from a poroid benign lesion. Herein, we report a case of PC arising from a hidroacanthoma simplex (HS), describing its dermoscopic features to highlight the possible usefulness of dermoscopy in identifying malignant changes.

## Case Presentation

**Figure 1:**
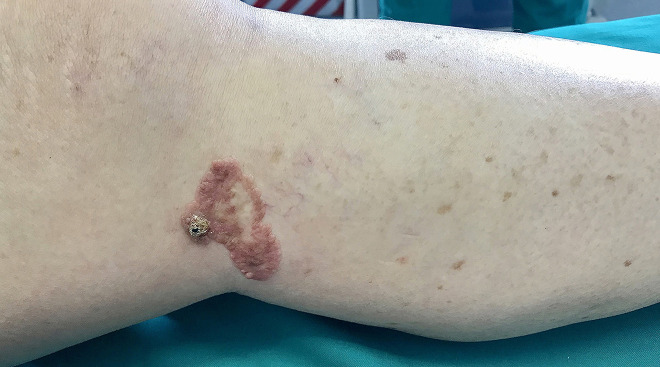
Clinical examination shows a well-demarcated plaque of 4.7 × 2.5 cm in size on the left leg, with a superimposed keratotic nodule at the periphery.

**Figure 2:**
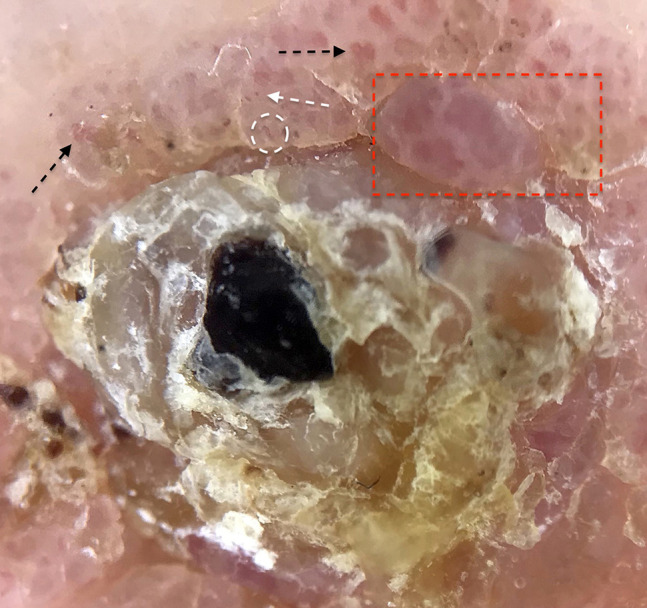
Polarized dermoscopy (×10) of the nodule reveals central crusting, faint/blurred polymorphous vessels (chalice-shaped [black arrow], dotted [white arrow], and linear [white circle]), and round-to-oval pink areas with different diameters surrounded by a peripheral white halo (box).

**Figure 3:**
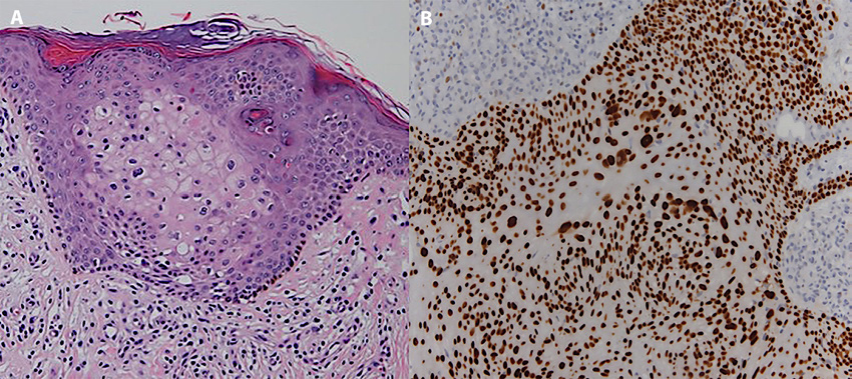
(A) Histology displays enlarged tumor cells with pale cytoplasm along with pleomorphic and hyperchromatic nuclei with mitoses but no evidence of dermis invasion (H&E, ×10). (B) Immunohistochemistry for p16 clearly marks the limits of porocarcinoma in relation to hidroacanthoma simplex (immunohistochemical stain for p16, ×10).

A 75-year-old Caucasian woman presented to our clinic for evaluation of an asymptomatic pinkish plaque on her left leg that had been biopsied several times and diagnosed as HS (Figure 1). At subsequent follow-up visit, a keratotic lesion compatible with PC was found. No lymphadenopathy was present. Dermoscopy of the nodule displayed crusts, whitish scales, and red oval areas of varying diameter surrounded by a white halo. Inside these lobules, various types of faint/blurred polymorphous vessels (chalice-shaped, dotted and linear) were present (Figure 2). A skin biopsy of the nodule, performed for histopathological assessment, revealed neoplastic intraepidermal proliferation of enlarged cells with pale cytoplasm arranged in clusters and pleomorphic and hyperchromatic nuclei with mitoses; no evidence of dermal invasion was present (Figure 3A). Immunohistochemistry showed positivity for CK5/6, p16, EMA, and CEA markers (Figure 3B). Based on the aforementioned histological and immunohistochemical findings, a diagnosis of PC in situ was made. A wide local excision of the lesion with histologically clear margins was carried out.

## Conclusions

To date, English literature offers only 9 histologically proven instances of PC arising from HS. In 2 of these cases, dermoscopic assessment was also performed and found to be useful to highlight possible malignant changes [1,2]. In particular, the polymorphism of vessels (including dotted, linear-irregular, glomerular, and hairpin vessels in one instance and glomerular and hairpin vessels in the other) was observed in the superimposed PC in both instances. Irregularly shaped whitish negative network and hemorrhages along with crusts/scales over pink-whitish or gray-whitish structureless areas were additional features in each case, respectively.

It is important to note that the polymorphous vascular pattern and round-to-oval pink areas surrounded by a peripheral white halo (corresponding to clusters of cancer cells surrounded by stroma) are the main dermoscopic features of PC, yet such findings may also be seen less commonly in eccrine poroma. Nevertheless, their presence may still be helpful in assisting possible malignant changes in HS as it typically shows different features, ie, brown globular areas surrounded by scaling collarette or white globular areas surrounded by regular brown lines.

Notably, in our case, vessels were quite faint and blurred owing to the intraepidermal localization of tumor with vessels arranged more deeply, yet they were still useful as they are not expected in HS. The same is valid for the presence of round-to-oval pink areas surrounded by peripheral white halos. It is interesting that, as mentioned above, these structures may also be seen in eccrine poroma but in this benign tumor they are typically uniform, while in PC they are inhomogeneous, probably because of irregular growth of cancer cell nests.

Dermoscopy may be a useful additional tool in detecting possible malignant changes in HS.
